# Hardware acceleration of genomics data analysis: challenges and opportunities

**DOI:** 10.1093/bioinformatics/btab017

**Published:** 2021-05-25

**Authors:** Tony Robinson, Jim Harkin, Priyank Shukla

**Affiliations:** School of Computing, Engineering and Intelligent Systems, Ulster University, Magee Campus, Derry/Londonderry, BT48 7JL, UK; School of Computing, Engineering and Intelligent Systems, Ulster University, Magee Campus, Derry/Londonderry, BT48 7JL, UK; Northern Ireland Centre for Stratified Medicine, Biomedical Sciences Research Institute, Ulster University, C-TRIC Building, Altnagelvin Area Hospital, Derry/Londonderry, BT47 6SB, UK

## Abstract

The significant decline in the cost of genome sequencing has dramatically changed the typical bioinformatics pipeline for analysing sequencing data. Where traditionally, the computational challenge of sequencing is now secondary to genomic data analysis. Short read alignment (SRA) is a ubiquitous process within every modern bioinformatics pipeline in the field of genomics and is often regarded as the principal computational bottleneck. Many hardware and software approaches have been provided to solve the challenge of acceleration. However, previous attempts to increase throughput using many-core processing strategies have enjoyed limited success, mainly due to a dependence on global memory for each computational block. The limited scalability and high energy costs of many-core SRA implementations pose a significant constraint in maintaining acceleration. The Networks-On-Chip (NoC) hardware interconnect mechanism has advanced the scalability of many-core computing systems and, more recently, has demonstrated potential in SRA implementations by integrating multiple computational blocks such as pre-alignment filtering and sequence alignment efficiently, while minimizing memory latency and global memory access. This article provides a state of the art review on current hardware acceleration strategies for genomic data analysis, and it establishes the challenges and opportunities of utilizing NoCs as a critical building block in next-generation sequencing (NGS) technologies for advancing the speed of analysis.

## 1 Introduction

In the 1990s, the human genome project created the first draft sequence of the entire human genome at an estimated cost of USD 3 billion (Muir *et al.*, 2016; Sboner, 2011). Since then, the cost of sequencing has been declining exponentially. The significant output of massively parallel next-generation sequencing (NGS) technologies has a compounding effect on many challenges across the bioinformatics pipelines (Lightbody *et al.*, 2019). Such technologies within the field of genomics have caused a shift in emphasis from sequencing as the principal challenge to efficient methods of accessing, sharing and analysing data (Lightbody *et al.*, 2019; McVicar *et al.*, 2016; Muir *et al.*, 2016). Personalized medicine, aims to make genomic medicine part of a standard battery of tests (Lightbody *et al.*, 2019). Readily available genomic data insights offer the promise of tailored prescription of treatment and ultimately, highly bespoke care (Brittain *et al.*, 2017). For the realization of the goals of personalized medicine and to be genuinely personal, genomic data insights must be accessible (Orth *et al.*, 2019).

In the field of genomics, short read alignment (SRA) is an essential component within the modern bioinformatics pipeline and is one of the most significant computational challenges to date (Lightbody *et al.*, 2019). Fundamentally a string matching problem, the complexity of read alignment arises from the sheer volume of raw genomic input data (Lightbody *et al.*, 2019; Muir *et al.*, 2016; Sboner, 2011). For perspective, the human genome is an estimated 3.2 billion characters long, with short read lengths typically containing 100-300 characters (Sboner, 2011). Thus, a search usually extends the full reference genome for each read resulting in billions of searches, making it computationally intensive (McVicar *et al.*, 2016). Previous attempts to increase read alignment throughput have included multistage alignment algorithms (McVicar *et al.*, 2016), pre-alignment filters (Kaplan *et al.*, 2019) and many-core processing (Liu *et al.*, 2017).

This article presents a review of the literature on the computational challenges of SRA and in particular, focuses on hardware acceleration strategies. Furthermore, it examines previously implemented NoCs as a mechanism to overcome the principle problem of memory accessibility that currently limits the scale of acceleration. Section 2 provides contextual background and introduce the process of SRA and a typical genomics study with bioinformatics pipeline. Sections 3 and 4 present the principle computational challenges and opportunities related to SRA, focusing on hardware acceleration and NoCs. Lastly, Sections 5 and 6 offer discussion and concluding thoughts on the information presented.

## 2 Genome sequencing and genomic data analysis

### 2.1 Next-generation sequencing (NGS)

NGS techniques are massively parallel, allowing for whole-genome sequencing at unprecedented scale and speed (Behjati and Tarpey, 2013; ThermoFisher Scientific, 2020).

**First generation**: Sanger sequencing, a dominant technology of the 70s and 80s, were enablers in realizing the human genome for the first time (Rizzo and Buck, 2012). Some estimates placed the cost of sequencing the human genome with Sanger sequencing at $10millon and $25million (Margulies *et al.*, 2005). The highly targeted chain termination method known as Sanger sequencing produce long reads (approx. 400 - 1000 bp), which in turn lends itself to the validation of NGS sequencing data due to its high accuracy (Kosuri and Church, 2014). Although expensive and labour intensive, Sanger sequencing created a demand for reliable high throughput sequencing at low cost (Rizzo and Buck, 2012).

**Second generation**: Pyrosequencing method commercialized by Roche/454 Life Sciences, sequencing-by-synthesis method commercialized by Solexa/Illumina and sequencing by oligonucleotide ligation and detection (SOLiD) method commercialized by ABI/Life Technologies represent the second generation of sequencing methods and beginning of NGS revolution. Shorter read lengths (35-700 bp) and high-throughput (1 million—2 billion) are the notable features of these methods. The chemistry behind these methods has been extensively reviewed elsewhere (Goodwin *et al.*, 2016). The pyrosequencing method maintained an average read length of 108 bp, now typically producing between 230-700 bp; the longest read length among second-generation sequencing technologies (Hasnain, 2020). Sequencing machines based on sequencing-by-synthesis and SOLiD methods boast a throughput in billions, especially the NovaSeq^TM^ 6000 system from Illumina claims to produce 3000 gigabases (Illumina Inc., 2019).

**Third generation**: Single Molecule Real-Time (SMRT) sequencing method commercialized by Pacific Biosciences, produces 100 - 200 gigabases per single 20-hour run, with approximately 30000 bp read lengths (Du *et al.*, 2019). Despite its high throughput, SMRT lacks the raw sequence accuracy of pyrosequencing at 87% compared to 99% (Du *et al.*, 2019). The cost per one million bases is $10 compared to approximately $2400 for pyrosequencing (Liu *et al.*, 2012). However, in recent years algorithms such as *LSCplus* (Hu *et al.*, 2016), *HybridSPAdes* (Antipov *et al.*, 2016), *HALC* (Bao and Lan, 2017) and *ReMILO* (Bao *et al.*, 2018) have been proposed improving the accuracy and reducing associated costs of SMRT assembly through overlap detection and misassembly detection.

**Fourth generation**: Nanopore sequencing method commercialized by Oxford Nanopore Technologies offers high consensus raw read accuracy of 99.96% at a comparative cost to SMRT sequencing methods (Hasnain, 2020). In addition, as nanopore sequencing is entirely library dependant, it can produce up to 500 kbp, with the longest read recorded at 2272580 bp (Hasnain, 2020; Payne *et al.*, 2019). The MinION system from Oxford Nanopore weighs <100 g and thus offers the portability for sequencing as-you-go in a real-time environment (Oxford Nanopore Technologies, 2020).

Modern multiplexing methods overcome a historical limitation of many first and second-generation sequencing technologies; that of requiring large volumes of input DNA. Particularly where investigations concern different target regions while additionally reducing runtime and associated costs (Fleckhaus and Schneider, 2020; Shang *et al.*, 2020). An extensive review of the evolution of NGS technologies has been covered elsewhere (Niedringhaus *et al.*, 2011).

### 2.2 Applications of genomic data

***De novo* assembly** typically refers to the development of a genome from which genomic data insights are gained without the presence of a reference genome (Lightbody *et al.*, 2019). *De novo* assembly relies on comprehensive deep sequenced and high coverage sample data to construct a genome (Ayling *et al.*, 2020; Ghurye *et al.*, 2016). Long reads are naturally more suited to *de novo* studies where the length of the read typically makes genome assembly easier (Turakhia *et al.*, 2019). Long reads are often associated with studies advocating reference-free variant calling (Croville *et al.*, 2018; Li, 2018; Turakhia *et al.*, 2019), discussed later in more detail.

**Metagenomics** is primarily concerned with the sequencing of an environmental sample for phenotype identification and quantitative analysis of various microorganisms (Ayling *et al.*, 2020). NGS applied to environmental samples rely on the availability of reference genome databases. The low coverage of most species in a sample renders *de novo* assemblies unviable (Ayling *et al.*, 2020; Ghurye *et al.*, 2016).

**Epigenomics** is an integral part of functional genomics, exploring reversible modifications to DNA that affect gene expression without altering the DNA sequence (Angerer *et al.*, 2017). Such modifications play a crucial role in gene expression and regulation (Angerer *et al.*, 2017; Chen and Snyder, 2013). The study of how proteins interact with DNA to regulate gene expression is essential to fully understand complex biological processes and disease states (Clark *et al.*, 2013; Joshi and Patil, 2017). Chromatin immunoprecipitation followed by sequencing (ChIP-seq), DNase I hypersensitive sites sequencing (DNase-seq) and formaldehyde assisted isolation of regulatory elements followed by sequencing (FAIRE-seq), among others, are used to determine such protein interactions (Park, 2009).

**Transcriptomics** represents the complete set of all the ribonucleic acid (RNA) molecules (Milward *et al.*, 2016). Therefore, transcriptomics covers all types of transcripts, including messenger RNAs (mRNAs), microRNAs (miRNAs) and different kinds of long non-coding RNAs (lncRNAs), including their transcription and expression levels, functions, locations and degradation (Milward *et al.*, 2016).

**Targeted resequencing** refers to the sequencing of a discrete genomic locus of an individual or population to detect variations between the individual or population and the standard genome of the species. It can be divided into: i) genotyping, i.e. testing for known mutations, and ii) variation analysis, i.e. scanning for any mutation or variants in a target genomic region. Variants are defined as single nucleotide variants (SNVs), small insertions and deletions (indels) and structural variants (SVs) (Bohannan and Mitrofanova, 2019).

**Variant calling** focuses on the identification of genetic variants at a whole genome or exome level from DNA sequencing data by comparing it to a known reference genome. For cases where no reference is available or consist of a high number of variants or poor quality sequence alignments, *de novo* assembly can recover genomic variation at the expense of computational resources (Audano *et al.*, 2018). In addition, approaches such as *Kestrel* (Audano *et al.*, 2018), *MALVA* (Denti *et al.*, 2019) and *MALVIRUS* (Ciccolella *et al.*, 2020) allow for reference-free variant calling *via* haplotype reconstruction from *k*-mer frequencies and known variants (Audano *et al.*, 2018; Denti *et al.*, 2019).

### 2.3 Typical bioinformatics pipeline

**Fig. 1.**
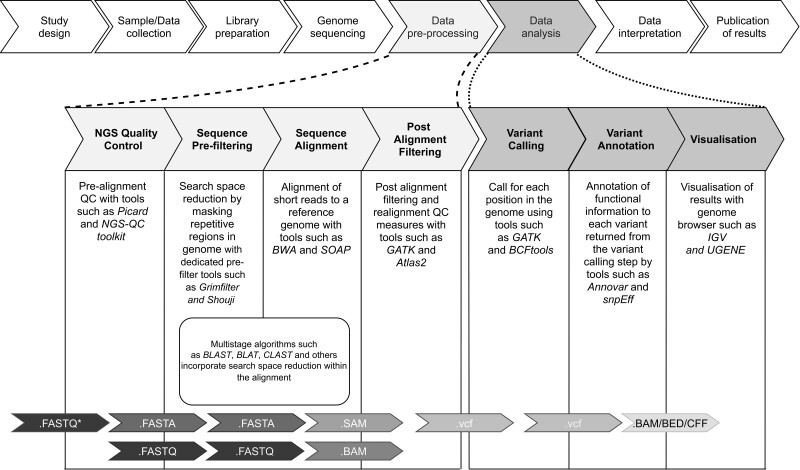
Typical variant calling bioinformatics pipeline composed of steps following NGS sequencing leading to the visualization of data is presented in the middle panel. The variant calling bioinformatics pipeline is contained within the data pre-processing and data analysis stages of a much larger bioinformatics-based research study as illustrated in the upper panel. Data file formats at each step are presented in the lower panel (Al Kawam *et al.*, 2017; Lightbody *et al.*, 2019). *Platform-specific raw sequence output either .BAM or .FASTQ or .HDF5 (NCBI, 2019).

A bioinformatics based research study typically consists of study design, sample collection, library preparation to eventual NGS sequencing and data analysis (Fig. 1, upper panel) (Lightbody *et al.*, 2019). Within which, a typical bioinformatics pipeline represents data pre-processing and data analysis workflows actioned to yield useable insights from sequenced samples. Such workflows are typically dependent upon the end application, such as variant calling (Fig. 1, middle panel), and thus, overall study design. However, they share some common steps such as quality control, alignment, pre- and post-alignment filtering and visualization. Each step has its own unique set of barriers and facilitating factors, which have an ultimate bearing on the quality of data output for analysis (Lightbody *et al.*, 2019).

**NGS quality control** (QC) is an integral part of the bioinformatics pipeline, one which ultimately determines the quality of insights achieved (Patel and Jain, 2012). Typically, early QC consists of sequence trimming, format conversions and QC statistics (Patel and Jain, 2012). Tools such as *Picard* (Broad Institute, 2015) and *NGS-QC toolkit* (Patel and Jain, 2012) provide a comprehensive suite of tools and workflows for QC and the generation of FASTQ files required for downstream analysis (Patel and Jain, 2012).

**Sequence pre-filtering** or pre-alignment filtering dramatically decreases the overall mapping time by identifying candidate locations for match and masking repetitive regions, thereby reducing the search space for alignment (Alser *et al.*, 2019). Dedicated pre-alignment filters, such as *Gatekeeper* (Alser *et al.*, 2017) *Shouji* (Alser *et al.*, 2019) and *grim-filter* (Kim *et al.*, 2018), are computational blocks available for implementation within hardware acceleration architectures.

**Sequence alignment** is the process of aligning short reads to a known reference genome, in order to generate a sequence alignment map (.SAM) file (Patel and Jain, 2012). This process typically consists of supplying a file containing the short reads and quality score (.FASTQ or .fq) (Patel and Jain, 2012) and a reference genome file (.FASTA or .fa) to a short read alignment algorithm, such as *BWA* (Li and Durbin, 2009), *Bowtie* (Langmead *et al.*, 2009a) or *SOAP* ([Bibr btab017-B73][Bibr btab017-B73]), which complete the mapping. The process of short read alignment is, as previously mentioned, a significant challenge for genomic data analysis and is the topic of this review. As such, various aspects of short read alignment and associated hardware acceleration are discussed in the following sections.

**Post-alignment filtering and realignment** QC measures, such as removing low quality or duplicate alignments are often implemented using *SAMtools mpileup* (Genome Research Ltd, 2020), *Genome Analysis Toolkit* (*GATK*) (Broad Institute, 2020) and *Atlas2* (Challis *et al.*, 2012). Local realignment is considered an alignment improvement step consisting of alignment quality control measures such as indel realignment and base quality score recalibration. It enhances the quality of alignment for regions of the mapping which either contain indels, mismatches or with lower coverage compared to the rest of the map (Tian *et al.*, 2016).

**Variant calling** is of primary importance to clinical practice and pharmacogenomics (Lightbody *et al.*, 2019). Traditional and benchmarking variant callers include *GATK* (Broad Institute, 2020), *Mapping and Assembly with Quality* (*MAQ*) (Li *et al.*, 2008b) and *SAMtools* (Li *et al.*, 2009a), among others. While *MAQ* and *SAMtools* are popular in practice, *GATK* is one of the oldest, most commonly used and a benchmark tool that has been extensively adapted by many bioinformatics pipeline developers (Goyal *et al.*, 2017). Regardless, all variant callers must solve the problem of distinguishing between legitimate mutations, experimental noise and sequencing error (Bohannan and Mitrofanova, 2019). As such, many algorithms concerned with a variant calling are multistage and have significant accuracy constraints to maximize clinical impact (Cardon and Harris, 2016; Ward *et al.*, 2013).

**Variant annotation and visualization** is an essential step for genomic data analysis where functional information is added to identified positions using tools such as *ANNOVAR* (Wang *et al.*, 2010) and *snpEff* (Cingolani *et al.*, 2012) and visualized using tools such as *UGENE* (Golosova *et al.*, 2014) and *integrative genomics viewer (IGV)* (Robinson *et al.*, 2011).

### 2.4 Short read alignment

The output reads from NGS machines lack any genome location (coordinates) information. Consequently, for meaningful insights, each read must be first mapped to a known reference genome (Lightbody *et al.*, 2019). This process is known as short read alignment (SRA), or mapping to reference (McVicar *et al.*, 2016). As Muir *et al.* (2016) suggest, sequence alignment is typically an early critical stage of a long bioinformatics pipeline. The complexity of modern high throughput sequence alignment is the challenge of comparing highly repetitive short read strings to a more extensive, equally repetitive reference string that is ∼ 3.2 billion characters (McVicar *et al.*, 2016). Short read alignment is, in essence, a string matching problem of vast scale in which two strings are compared and scored based on dissimilarity (Doan *et al.*, 2012). Many computational tools have been introduced to facilitate sequence alignment and are discussed in greater detail in the subsequent sections.

Edit distance is the primary calculation metric used to quantitatively measure dissimilarity between two sequences (Fei *et al.*, 2018). Thus, it is fundamental within SRA and typically implemented *via* the Levenshtein or Hamming distance calculation (Zokaee et al., 2018). Hamming distance is defined between two sequences of equal length, where the returned value is the number of positions with a mismatch (Doan *et al.*, 2012). Conversely, Levenshtein distance does not require two sequences of equal length and returns the minimal number of edit operations required to change one sequence to another (Doan *et al.*, 2012). Such edit operations are defined as insertion, deletion and mismatch, i.e. alteration of a single character in either sequence (Doan *et al.*, 2012). Edit distance is often implemented using a generalized form of Levenshtein distance, such as the Needleman-Wunsch (NW) algorithm or Smith-Waterman (SW) algorithm (Doan *et al.*, 2012). Such methods represent pairwise sequence alignment, which aligns two sequences either *via* global or local alignment, typically producing a highly accurate and exhaustive alignment. Global alignment aligns two sequences base-by-base from one end to the other such as the NW alignment algorithm (Li and Wren, 2014). Local alignment, aligns sub-sequences of two sequences, based upon highest similarity matching, for example, the SW algorithm (Banerjee *et al.*, 2019).

While edit distances measure the dissimilarity of two sequences, in molecular biology, it is common to define scores as measures of sequence similarity (Lesk, 2008). Algorithms for finding optimal alignment, such as dynamic programming (DP), can seek either to minimize a dissimilarity measure or to maximize the scoring function (Lesk, 2008).

Computation of the two dimensional DP matrix for finding the optimal pairwise sequence alignment(s) between the two sequences consists of four distinct steps: i) defining the scoring schema, ii) initializing the boundary conditions for top row and left column of the matrix, iii) populating the matrix using an update function and finally iv) backtracking to highlight the optimal alignment(s) (Al Kawam *et al.*, 2017; Lesk, 2008).

- **Defining the scoring schema**: A penalty or cost function is an arbitrary integer assigned for the match, mismatch and insertion or deletion represented as Δ (Banerjee *et al.*, 2019; Lesk, 2008), and generally expressed as:
  1 $$\Delta \left(Q_{i},R_{j}\right)\;=\;\Delta \left(\mathrm{match}\right)\;if\;Q_{i}\;=\;R_{j}$$
   
  2 $$\Delta \left(Q_{i},R_{j}\right)\;=\;\Delta \left(\mathrm{mismatch}\right)\;if\;Q_{i}\;\neq \;R_{j}$$
   
  3 $$\Delta \left(\Phi ,R_{j}\right)\;=\;\Delta \left(Q_{i},\Phi \right)\;=\;\Delta \left(\mathrm{delete}\right)\;=\;\Delta (\mathrm{insert})$$
  where Q and R are two input strings of length m and n, respectively, $\left(\Phi ,R_{j}\right)$ represents deletions in Q or insertions in R and $\left(Q_{i},\Phi \right)$ correspond to insertions in Q or deletions in R and Δ is the cost function or penalty associated with edit operation. (Al Kawam *et al.*, 2017; Lesk, 2008).
  An alternative and more sophisticated method of imposing penalty scores is an affine gap penalty model, which distinguishes between the cost of opening a gap and the cost of continuing a gap rather than applying a fixed penalty for gaps greater than 1 bp in length (Doan *et al.*, 2012). The model assigns $C_{o}+\left(k-1\right)C_{r}$ to each gap of length k where $C_{o}$ is the cost of opening a gap, $C_{r}$ the cost of continuing, such that $C_{r}<C_{o}$ (Doan *et al.*, 2012). For simplicity of explanation of the alignment process, we have focused on constant gap penalty model here.
- **Initialization of boundary conditions in DP matrix**: For NW global alignment, following the scoring schema expressed in Equations 1, 2 and 3, the gap penalty conditions are imposed in the top row and leftmost column while initializing the first cell within the matrix, by deploying the Equations 4 and 5 (Lesk, 2008).
  4 $$S\left(i,0\right)\;=\;\sum_{k=0}^{i}\Delta \left(Q_{k},\Phi \right)\;\mathrm{for}\;0\leq i\leq m\;$$
   
  5 $$S\left(0,j\right)\;=\;\sum_{k=0}^{j}\Delta \left(\Phi ,R_{k}\right)\;\mathrm{for}\;0\leq j\leq n$$
  For SW local alignment, top row and leftmost column of the DP matrix are usually set to a fixed value following the Equations 6 and 7 (Al Kawam *et al.*, 2017).
  6 $$S\left(i,0\right)\;=\;\mathrm{boundary}\;1\;\mathrm{value}\;0\leq i\leq m$$
   
  7 $$S\left(0,j\right)\;=\;\mathrm{boundary}\;2\;\mathrm{value}\;0\leq j\leq n$$
- **Populating the DP matrix**: Following initialization, each cell in S is updated according to the recurrent relationship expressed in Equation 8 (Al Kawam *et al.*, 2017).
  8 $$S\left(i,j\right)\;=\;\max\left\{\begin{array}{c} S\left(i,j-1\right)+\Delta \left(\Phi ,R_{j}\right)\;\\ S\left(i-1,j-1\right)+\Delta \left(Q_{i},R_{j}\right)\\ S\left(i-1,j\right)+\Delta \left(Q_{i},\Phi \right) \end{array}\right\}$$
  where $Q_{i}$ represents the base in position i of first sequence Q and $R_{j}$ represents the base in position j of second sequence R, $S\left(i-1,j-1\right)+\Delta \left(Q_{i},R_{j}\right)$ corresponds to a match between $Q_{i}\;\mathrm{and}\;R_{j}$ or a mismatch leading to substitution $Q_{i}↔R_{j}$, $S\left(i,j-1\right)+\Delta \left(\Phi ,R_{j}\right)$ inserts a gap in the sequence $Q_{i}$ and finally $S\left(i-1,j\right)+\Delta \left(Q_{i},\Phi \right)$ inserts a gap in the sequence $R_{j}$.
- **Backtracking to highlight the optimal alignment(s)**: In the case of NW global alignment, the optimal alignment score is achieved in only the lower-right cell of the DP matrix. Therefore, optimum alignment is recovered by tracing a path back through the matrix from $\left(m,n\right)$ to (0,0) indicating all the possible optimum alignments (Lesk, 2008). In the case of SW local alignment, the optimal alignment score is the maximum score which can be encountered anywhere in the matrix. Therefore, optimum alignment is recovered by tracing a path back from that particular cell, and it continues only as far as the region of local similarity continues (Lesk, 2008).

**Fig. 2.**
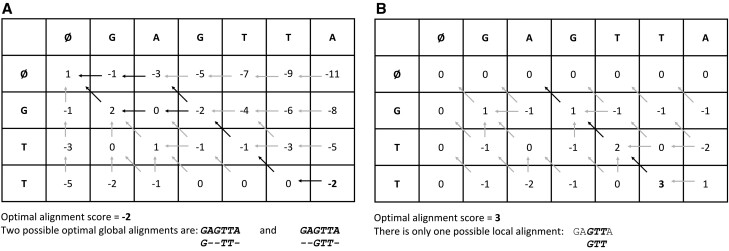
(**A**) Global and (**B**) local alignment of two sequences Q=GTT and R=GAGTTA, with scoring schema $\Delta \left(\mathrm{match}\right)=+1,\;\Delta \left(\mathrm{mismatch}\right)=-1,\;\Delta \left(\mathrm{insert}\right)=\Delta \left(\mathrm{delete}\right)=-2$. Optimal alignment scores are highlighted in bold font and paths for tracing back the optimal alignments are highlighted in bold arrows. Note the gaps in (A) appearing outside the matched regions, leading to global alignments. No gaps appear in (B) outside the matched region leading to a local alignment.

Consider an example whereby two sequences Q=GTT and R=GAGTTA are aligned as per NW global alignment (Fig. 2a) and SW local alignment (Fig. 2b) strategies. Scoring schema is set as:

$$\Delta \left(\mathrm{match}\right)=+1,\;\Delta \left(\mathrm{mismatch}\right)=-1,\;\Delta \left(\mathrm{insert}\right)=\Delta \left(\mathrm{delete}\right)=-2$$

Following the above scoring schema and Equations 4 and 5, the matrix for NW global alignment is initialized for top row and leftmost column, and populated from the recurrence relationship defined in Equation 8 (Fig. 2a). For SW local alignment, the DP matrix is initialized with a constant boundary value of 0 for both top row and leftmost column following Equations 6 and 7, and populated from the recurrence relationship defined in Equation 8 (Fig. 2b). In the case of NW global alignment, optimal alignment score always appears in the lower-right column; hence here it is -2 (Fig. 2a). There are two possible global alignments with the same optimal score of -2, backtracked in bold arrows (Fig. 2a). In the case of SW local alignment, optimal alignment score is the maximum score which can appear anywhere in the matrix; hence here it is 3 (Fig. 2b). There is only one local alignment with an optimal score of 3, backtracked in bold arrows (Fig. 2b).

## 3 Computational challenges of short read alignment

Sboner (2011) highlighted the changing computational priorities, estimating downstream analysis and experimental design becoming principal problem areas, compared to fifteen years ago, where the most significant bottlenecks were associated with gene sequencing. The following section outlines the current challenges in SRA and their memory intensive nature.

### 3.1 Alignment challenges

The dominant alignment paradigm, pairwise sequence alignment, produces optimal exhaustive alignments either *via* global or local alignment at the expense of speed and power consumption (Muir *et al.*, 2016). Although both alignments are accurate, such pragmatic short read alignment makes it impossible to map sequences to large reference genomes due to quadratic complexity; where the time taken to process the data grows exponentially when the data input increases linearly (Muir *et al.*, 2016).

According to Banerjee *et al.* (2019), edit distance computation in short read alignment typically dominates 50% - 70% of the runtime. To illustrate this further, the SRA algorithm *SNAP* (Zaharia *et al.*, 2011) calls the edit distance 51 times per read. There has been extensive research involving index strategies to reduce the number of candidate locations requiring calculation (Alser *et al.*, 2019; Banerjee *et al.*, 2019). As such, a large proportion of alignment algorithms have been designed to pre-filter alignment candidate locations before Levenshtein distance calculations as a means to reduce the search space (Banerjee *et al.*, 2019).

Multistage heuristic algorithms such as *BLAST* (Altschul *et al.*, 1990), *MAQ* (Li *et al.*, 2008a) and *STAR* (Dobin *et al.*, 2013) use hash and index lookups to identify promising location data, and then scan for a match typically using a Smith-Waterman stage aligner (McVicar *et al.*, 2016). As such, they usually are much faster at alignment and more flexible than exhaustive DP algorithms; although they deliver sub-optimal results (McVicar *et al.*, 2016; Muir *et al.*, 2016). Table 1 below provides a comparison between some popular SRA algorithms.

**Table 1.** CPU based alignment algorithms and their critical performance metrics.

Algorithm	Performance features			Basic features			References
	Speed (reads/sec)	Reads aligned (%)	Memory footprint (GB)	Min read length (bp)	Max read length (bp)	Compression method	
*BLAT*	185	95.0	3.8	11	5000000	–	Kent (2002) and Fonseca *et al.* (2012)
*Bowtie*	5556	79.9	5.0	4	1024	FM-index	Langmead *et al.* (2009a) and Fonseca *et al.* (2012)
*Bowtie2*	2083	99.2	5.1	4	5000000	FM-index	Fonseca *et al.* (2012) and Langmead and Salzberg (2012)
*BWA*	1282	92.8	7.6	4	200	BWT	Li and Durbin (2009) and Fonseca *et al.* (2012)
*MAQ*	51^*^	97.4^*^	1.0^*^	28	63	Hash table	Li *et al.* (2008b)
*SNAP*	37000^†^	94.0^†^	1.2^†^	–	–	Hash table	Zaharia *et al.* (2011)
*SOAP2*	4167	79.9	5.3	27	1000	BWT	Li *et al.* (2009b) and Fonseca *et al.* (2012)
*STAR*	2083^‡^	94.0^‡^	2.3^‡^	–	>1000	Suffix arrays	Dobin *et al.* (2013)

*Note:* Performance features, where unless stated otherwise, are based upon the alignment of 1 million, 100 bp, single-end reads with the human genome (*Homo sapiens*, assembly GRCh37) on a single-core CPU, with 32 GB of RAM. Speed (reads/sec) is the number of reads aligned to the reference genome per second. Reads aligned (%) is the percentage of reads aligned to the reference genome. Memory footprint is the quoted operational peak memory usage (GB) per processing core. Min and Max read length (bp) are the reported read lengths that can be aligned. The compression method is the algorithm used by the aligner for reference genome compression. The information which is not obtainable is denoted as (–). Please refer to the respective article(s) mentioned in the table for further details.

^*^MAQ performance features are based upon mapping of 100 million, 35 bp, paired-end reads. Computing hardware specifications are unavailable.

^†^SNAP performance features are based upon mapping of 100 million, 125 bp, single-end reads. SNAP benchmarking, as reported by Zaharia *et al*. (2011) is based on a 256 GB RAM computing system.

^‡^STAR performance features are based upon mapping 10 million, 76 bp, paired-end reads. STAR benchmarking, as reported by Dobin *et al*. (2013) is based on a 148 GB RAM computing system.

### 3.2 Hardware acceleration

There are several key hardware platforms that support the acceleration of sequence alignment algorithms, as discussed below.

**High-performance computing (HPC) cluster** is a series of interconnected desktop computers with central processing units (CPUs) or network servers linked together to form a computing array, typically in a ‘master-mason’ configuration (Hackl *et al.*, 2014; Langmead *et al.*, 2009b). A specified computer acts as the user interface to the rest of the network. The remaining machines within the system carry out computational tasks as defined by the master computer. This configuration has gained popularity due to the low cost and low barrier to entry for small to medium laboratories using standard hardware and software (Ben Abdallah, 2017; Lightbody *et al.*, 2019; Sboner, 2011). Despite the relative accessibility of HPC, the technical expertise required in-house for setup and bespoke maintenance of software applications running on the cluster is a limiting factor (Lightbody *et al.*, 2019). Open-source software frameworks such as *Apache Hadoop* support the scheduling of parallel operations across the network to manage computational load (Lightbody *et al.*, 2019). Furthermore, *MapReduce*, a popular parallel programming framework by Google, has increased popularity within the genomic data processing literature as a means to facilitate SRA within a computing cluster (Al-Absi and Kang, 2015; Jourdren *et al.*, 2012; Schatz, 2009). As noted by Lightbody *et al.* (2019), *MapReduce* concepts have been implemented in other parallel solutions specific to genomic data processing such as the *GATK* (Goyal *et al.*, 2017; Lightbody *et al.*, 2019; Lv *et al.*, 2016).

**Cloud computing cluster** is similar to an HPC except, rather than connected *via* a local area network (LAN), computing nodes are connected remotely usually over the internet (Jackson *et al.*, 2010; Lightbody *et al.*, 2019; Schatz, 2009). One of the significant advantages of cloud computing is that they are highly scalable, on-demand and without the barrier of the in-house deployment of fixed computational resources (Lightbody *et al*., 2016). As such, recent years has seen a surge in online vendors offering high-performance cloud computing as a service, providing accessibility of high-performance computing to researchers (Jackson *et al.*, 2010; Lightbody *et al.*, 2019). While an internal cloud network might be more suitable for sensitive information, public clouds such as Amazon Web Services (AWS) are a viable option if data is anonymised and encrypted beforehand (Jackson *et al.*, 2010; Lightbody *et al.*, 2019).

**Graphics processing units (GPUs)** are high performance integrated circuits first proposed for graphic processing in 1973 (Barron and Glorioso, 1973). However, It was not until 1991 upon the release of the PlayStation one (PS1) by Sony and Toshiba that the GPU became a mainstream technology (Peddie, 2020). Like field-programmable gate arrays (FPGAs), GPUs offer a high degree of parallelism with more than 1000 fine-grained processing cores (Sundfeld *et al.*, 2017). Within the scope of genomic analysis, Sundfeld *et al.* (2017) demonstrated a GPU approach up to 24 times faster than a 16-core CPU solution for RNA alignment using the *CUDA-Sankoff* sequence alignment algorithm (Sundfeld *et al.*, 2017). The high performance of GPUs, however, results in considerable power consumption compared to FPGAs (Yano *et al.*, 2014). Despite this, GPUs are popular within high-performance computing and particularly within bioinformatics due to the relative ease at which a designer may implement an already existing short read alignment algorithm such as *BWA* (Fei *et al.*, 2018; Houtgast *et al.*, 2018).

**Field programmable gate array (FPGAs)** (Xilinx, 1999) are a programmable logic device consisting of an array of configurable logic blocks enabling both fine and coarse-grained parallelism of an algorithm to be exploited. Thus, enabling faster execution or acceleration with lower energy costs than HPCs. Each block is comprised of memory and computational units (Lightbody *et al.*, 2019). The significant advantages and principal reasons behind their surge in popularity for hardware acceleration are their intrinsic parallelism, re-programmable nature, significant flexibility for acceleration across many applications and low cost (Lightbody *et al.*, 2019). A principal barrier to the use of FPGAs is the requirement for technical expertise; however, more recent high-level design synthesis tools are addressing this issue (Lightbody *et al.*, 2019). The flexibility afforded by FPGAs also supports scalability, similar to cloud and computing clusters. In addition, FPGAs allow for the inclusion of dedicated computational blocks, such as pre-alignment filters (Kim *et al.*, 2018). A limitation compared with cloud and computing clusters is the interoperability of algorithms for FPGA deployment, as many SRA algorithms are designed for CPUs realizations, utilizing concepts such as hyperthreading to increase throughput (Wang and Wang, 2019).

### 3.3 Many-core processing and NoC interconnect

Due to the significant volume of data involved in sequence alignment, a stark relationship exists between scalability and speed of execution. The reliance on global memory access results in a substantial increase in system delays as the system scales in both volumes of data and processing cores. To successfully address the issue of scalability with the ability to maintain a high throughput of data from memory to processing cores, researchers such as Nsame *et al.* (2014) and Sarkar *et al.* (2010) explored the use of the Networks-on-Chip (NoC) interconnect strategy in many applications.

**Fig. 3.**
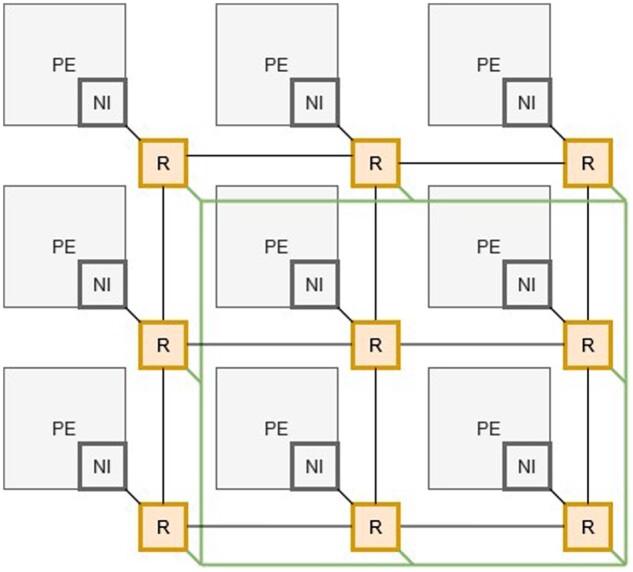
NoC mesh and ring topology adapted from Das and Ghosal (2018). PE, processing element; NI, network interface; R, switching router.

To appropriately convey the significance of NoC enabled hardware acceleration, it is necessary to illustrate the core components of an NoC which is comprised of three distinct physical parts (Fig. 3); network interface (NI) that connects the individual processing element (PE) to the switching router (R). The overall structure is referred to as a topology; for example, Figure 3 depicts a 2D array of interconnected nodes. Packets of data are communicated around the topology from source to destination processing element using the networks of routers. A routing algorithm is embedded within each router which reads the packet to obtain its origin and its destination node(s) and provides a direction (pathway) for the packet to travel in its destined journey. Given the multiple parallel links between routers, many packets can be communicated simultaneously, enabling a high throughput of data to be achieved *via* the use of multiple parallel paths with multiple packets. A comprehensive review of NoC structures and design is given by Tsai *et al.* (2012).

In addition, Subbulakshmi and Balamurugan (2014) provide a detailed analysis of NoC architectures of many-core systems-on-chip processing. The key benefit of NoC is the ability to scale in size (of processing elements that can be connected) while maintaining high levels of data throughput across the NoC structure. This has the impact of enabling acceleration to be maintained when a high frequency of data sharing among processing elements is required. The broad attributes of any NoC include the topology, routing algorithm and arbitration schemes. These combined together define an NoC and are explored in the design of NoC-based computing systems.

Early many-core alignment and NoC implementations such as Subbulakshmi and Balamurugan (2014) and Das and Ghosal (2018) demonstrated NoCs as an enabling mechanism that significantly increases the performance of hardware-based genomic data analysis. However, the correlation between the number of processing cores and transmission delays within the network poses a significant barrier to the implementation of NoC for SRA (Wang and Wang, 2019). Therefore, there exists an optimum network size depending on the alignment algorithm used; e.g. *BWA* scales linearly, whereas *HISAT2* (Kim *et al.*, 2019) shows a decay in execution speed with higher than 4 x 4 network sizes. This is further illustrated by Das and Ghosal (2018) who suggest that the NoC network topology, particularly those relying exclusively on mesh topologies, result in higher latency (slower performance) at higher network dimensions. Joardar *et al.* (2019) among others, identified significant routing constraints with NoCs for genomic data analysis, and add that read alignment algorithms require repeated memory accesses resulting in idle computation units and high latency times (Joardar *et al.*, 2019). As such, the traffic patterns produced are highly irregular. Many ‘off-the-shelf’ routing algorithms assume uniform traffic patterns and therefore are not suitable for *k*-mer counting or sequence alignment at large (Joardar *et al.*, 2019; Subbulakshmi and Balamurugan, 2014). This is echoed by Turakhia *et al.* (2017) who advocate the importance of co-design of software and hardware (Turakhia *et al.*, 2017, 2019). A key challenge in exploiting NoC is tailoring the routing algorithm for the application's traffic profile to minimize system latency and therefore, increase the throughput of data transmission (Liu *et al.*, 2016). Complexity arises from the dependency between the topology, arbitration scheme and routing algorithm in the tailoring exploration process.

## 4 Opportunities in short read alignment acceleration

There has been significant progress in recent years, combining many of the algorithms available to form hybrid SRA algorithms as previously discussed in Section 3. Furthermore, considerable effort has been made in exploiting the overlap between the various implementation technologies such as CPU clusters, GPU, cloud computing and FPGA hardware accelerators (Lightbody *et al.*, 2019). Techniques such as filtering and prefetching have been used to assist in accelerating the speed of computational operations in hardware (Alser *et al.*, 2019). In addition, the use of *MapReduce* frameworks in hardware and cluster implementations (software) with lossless compression methodologies, have attempted to bring the unfathomable data quantity to more manageable proportions (Al-Absi and Kang, 2015; Jourdren *et al.*, 2012). Table 2 provides a comparison between the four key hardware acceleration approaches representing the current state-of-the-art; *AligneR* (Zokaee *et al.*, 2018), *ASAP* (Banerjee *et al.*, 2019), *FPGASW* (Fei *et al.*, 2018) and *Darwin* (Turakhia *et al.*, 2019). The technologies chosen have significantly improved the speed of sequence alignment and serve to illustrate the computational challenges and opportunities discussed in this review.

**Table 2.** Comparison summary of four different hardware accelerators for sequence alignment.

Features	*AligneR* (Zokaee *et al.*, 2018)	*FPGASW* (Fei *et al.*, 2018)	*Darwin* (Turakhia *et al.*, 2017, 2019)	*ASAP* (Banerjee *et al.*, 2019)
Speed (reads/sec)	483k^*^	–	23k^†^	∼10k^§^
Max read length (bp)	1024	–	10k	128
Data structure	FM-index	–	–	–
Hardware accelerator processor	ReRam (specialist)	Xilinx Virtex-7 XC7VX485T FPGA	Xilinx Kintex-7 FPGA^‡^	Xilinx Virtex-7 XC7VX690T FPGA
Operating frequency (MHz)	100	200	250	250
Processing elements (PE) per array	–	512	64	256
GCUPS	–	105.9	–	609.6
Data bus	–	–	NoC interconnect	Crossbar
External memory (DRAM)	No external memory dependence	3 x 8GB DDR3-1600	4 x 32GB LPDDR4	–
Host CPU	–	Intel i5	Intel Xeon E5-26200	IBM power8
Host memory (GB) (DDR3 RAM)	–	8	64	–
Host interface	–	SFP+ Optical interface	×16 PCIe 2.0	CAPI interface
Search space reduction	–	–	D-SOFT	–
Edit distance function	Hamming	Levenshtein	–	Levenshtein
Gap penalty model	–	Affine	Affine	Constant
Edit distance implementation	Process-In-Memory (PIM)	Sequential logic	Sequential logic	Sequential logic
Power consumption (W)	1.9	44	15	6.9

*Note*: Speed is quoted in reads per second for simulated reads. Maximum read length (bp) is the reported maximum read length that can be aligned. Data structure corresponds to the compression mode utilized. Hardware accelerator processer is the main accelerator device used. Operating frequency (MHz) is the clock frequency of the accelerator hardware. Processing elements (PE) is the number of computational cells per dynamic programming (DP) matrix/array. GCUPS (Giga Cell Updates Per Second) is a performance measure of the number of processing element cell updates per second for a single array cell. Data bus is the interconnection strategy used. External memory (GB) corresponds to the available DDR3 RAM required to support accelerator operation. Host CPU is the CPU of interface computer to the accelerator. Host memory (GB) is the memory capacity of the host computer which the accelerator can draw upon. Host interface is the communication interconnect between host and accelerator. Search space reduction corresponds to the search space reduction strategy used in the pre-alignment filtering stage. Edit distance function corresponds to the specific edit distance calculation method used. Gap penalty model corresponds to the specific gap (insertion or deletion) penalty method used for each implementation. Edit distance implementation is the mode in which each accelerator computes the edit distance function to determine optimum alignment. Power consumption (W) is the power consumed by the accelerator during alignment. The information which is not obtainable is denoted as (–). Please refer to the respective article(s) mentioned in the table for further details.

^*^ *AligneR* computing speed is based upon 10 million, 100 bp simulated short reads from human genome reference hg19.

^†^ *Darwin* computing speed is based upon 3 million, 1000 bp simulated short reads from human genome reference GRCh38.

^‡^Details on the actual device used in the case of *Darwin* are unavailable other than the Kintex-7 series by Xilinx.

^§^ *ASAP* computing speed is based upon 100 million, 128 bp simulated short reads from human genome reference hg38.

### 4.1 Alignment computation

Levenshtein distance calculations are typically performed sequentially on CPU by most alignment algorithms limiting data throughput. Notably, *AligneR* and *ASAP* have shown a considerable acceleration in their computation through the implementation of different dedicated hardware (Banerjee *et al.*, 2019; Zokaee *et al.*, 2018). *AligneR* uses specialized ReRAM devices instead of logic blocks where ReRAM modules are set to logic one and reset to logic zero, corresponding to different alignment scores as per the Hamming distance calculation (Zokaee *et al.*, 2018). *ASAP* implements Levenshtein distance calculations in sequential logic using FPGAs in which parameters are coded into clock cycle delays and operators to logic gates (Banerjee *et al.*, 2019). Both methods demonstrate reduced power consumption and higher throughput (Table 2).

### 4.2 Search space reduction

*Darwin* utilizes a novel algorithm known as *D-SOFT* (Turakhia *et al.*, 2017). *D-SOFT* uses large bins (i.e. ranked containers for candidate locations) covering 9 bp each, therefore composed of 18 bits (Turakhia *et al.*, 2017). *AligneR* uses variants of FM index pre-alignment and compression (Fei *et al.*, 2018; Zokaee *et al.*, 2018). *ASAP* and *FPGASW* do not disclose the search space reduction strategy used. Instead, they discuss such approaches within the context of alignment, therefore adopting an ‘alignment as a filter’ approach (Banerjee *et al.*, 2019).

### 4.3 Latency and memory overhead

Efforts to increase speed through closely coupling memory and computation, i.e. physically stacking computational blocks used for alignment with dedicated RAM has resulted in decreased accuracy, high energy consumption and high implementation costs (Liu *et al.*, 2017). As such, further scale in this regard produces diminishing returns. Therefore, memory overhead and memory accessibility are universal critical barriers to increasing speed of execution (Fei *et al.*, 2018). Turakhia *et al.* (2019) have illustrated the latency associated with random memory access patterns inherent within SRA as a potential point of acceleration.

*Darwin* produces 16GB of memory overhead from seed position tables stored in external memory with each processing element (PE) contributing 2kB per reading for storage in on-chip SRAM (Turakhia *et al.*, 2019). In addition, its 4 x 32GB DDR4 DRAM module contains copies of each of the alignment tables, thus balancing and optimising memory access with each DRAM module loading up to 4 seeds per cycle (Turakhia *et al.*, 2019). Overall, *Darwin* reports 15x speedup from memory optimization; 3x from reduced random access to DRAM (prefetching required data from SRAM) and 5x from changing the random access pattern to near sequential (Turakhia *et al.*, 2019). Similarly, *ASAP* used a modified shift register as part of the processing array to expand memory bandwidth and support larger reference tables for implementing more dynamic gap penalty models (Banerjee *et al.*, 2019). However, it does not include memory optimization, instead focuses on larger input data strings (Banerjee *et al.*, 2019).

*AligneR* bypasses memory latency bottlenecks entirely by adopting a process-in-memory (PIM) methodology (Zokaee *et al.*, 2018). Combined with an FM index compression strategy, this results in lower search space and memory overhead for associated indexing (Arram *et al.*, 2017). *AligneR*, unlike *ASAP* and *Darwin* dynamically switches between active process elements (PEs) within the array due to the short ReRAM cell endurance (Zokaee *et al.*, 2018). The mechanism adopted by *AligneR* potentially limits its scalability; as six error-correcting pointer tables are required for switching and reducing diagonal processing space (Zokaee *et al.*, 2018). Interestingly, *Darwin* utilizes Networks-on-Chip (NoC) interconnect for data transfer instead of a crossbar (Turakhia *et al.*, 2019).

### 4.4 Advances using Networks-On-Chip

The dependence on large external memory significantly limits scalability as the operational cost rises with the number of computational units (Sarkar *et al.*, 2010). However, the use of NoCs has shown potential in mitigating this dedicated RAM dependency through the intelligent management of global and local data memory access (Das and Ghosal, 2018). Initially proposed for short read alignment by Sarkar *et al.* in 2010, NoC-based hardware demonstrated a 2.5 x 10^4^ (reads per second) increase in speed compared to traditional CPU based alignment. Sarkar *et al.* (2010) argued that NoC-based implementations offer increased flexibility and further integration of computational elements within a chip. Wang and Wang (2019) further demonstrated the acceleration of popular computational algorithms using a novel NoC-based accelerator. Thus, the NoC paradigm provides a practical interconnection mechanism for enabling high integration of many-core designs with a high degree of modularity and explicit data-parallelism (Das and Ghosal, 2018; Joardar *et al.*, 2019; Sarkar *et al.*, 2010; Wang and Wang, 2019).

## 5 Discussion

As Muir *et al.* (2016) suggested, challenges associated with genome sequencing have been replaced with computational challenges related to downstream analysis (Muir *et al.*, 2016; Sboner, 2011). Efficient management, alignment, lossless compression and sharing of data with emphasis on security and privacy are now the dominant challenges of the modern bioinformatics pipeline (Lightbody *et al.*, 2019). SRA is a fundamental step in genomics data analysis and is, therefore, commanding in the overall efficiency of the analysis pipeline. SRA is perhaps one of the most significant challenges as the volume of data generated through genome sequencing continues to rise exponentially (Sboner, 2011). Thus, SRA efficiency is crucial to enable a balanced and robust bioinformatics pipeline as data requirements grow.

FPGAs are aptly suited to addressing these challenges due to their inherent fine and coarse-grained parallelism and flexibility (Lightbody *et al.*, 2019; McVicar *et al.*, 2016). Therefore, FPGA implementations, such as those demonstrated by Arram et al. (2013), Chen *et al.* (2014), McVicar *et al.* (2016) and more recently Gök *et al.* (2018) and Banerjee *et al.* (2019) have shown considerable promise of efficiently accelerating SRA in FPGA hardware. The majority of SRA algorithms are designed for CPU, cluster and cloud computing, utilizing concepts such as hyperthreading and linear task management (Shang *et al.*, 2014). As such, they do not take advantage of the fine-grained parallelism offered by FPGAs (Alser *et al.*, 2017). Alser et al. (2017), Kim *et al.* (2018) and Joardar et al. (2019) argue the need for co-design; the design of the hardware acceleration and SRA software in parallel. This is especially true at scale, with more varied computational blocks included within the system (Liu *et al.*, 2017). Implementations such as *ASAP* (Banerjee *et al.*, 2019) and *AligneR* (Zokaee *et al.*, 2018) sufficiently illustrate this requirement in which edit distance computation is executed as a systolic array in parallel within dedicated electronic hardware rather than sequentially on CPUs.

Interestingly, what Liu *et al.* (2017) identified with many-core implementations is that the dependence on global data RAM access with irregular traffic patterns prevent scalability and efficient use of the systems-on-chip resources (Liu *et al.*, 2017). Thus, this establishes the need for more adaptive and intelligent on-chip communications architectures (Subbulakshmi and Balamurugan, 2014; Wang and Wang, 2019). Sarkar *et al.* (2010) were perhaps the first to demonstrate the application of NoCs as a means to overcome this scalability issue. Later Das and Ghosal (2018) and Wang and Wang (2019) demonstrated the potential of NoCs as a means to enable scalability and efficient management of on-chip resources, removing the dependence on global memory through intelligent routing and arbitration.

Wang and Wang (2019) stipulated that the latency of the NoC results from transmission and computation times, where transmission time becomes exacerbated upon data packet congestion. As such, they proposed a bufferless mesh/ring network topology, whereby the ring network acts as an overflow in the event of congestion, providing a simple alternative path for data packets. They utilize a basic and standard non-adaptive XY routing algorithm within their proposal. A comparison between different routing algorithms is given by Sharifi *et al.* (2013). These works establish the need for adaptive routing algorithms and illustrate the complexity in balancing the goal of designing an effective adaptive routing algorithm, while ensuring it does not limit scalability due to large hardware area overheads.

In addition, Bahrebar and Stroobandt (2016) provide further details where NoC routing algorithms are explored within the scope of many-core design. This is a crucial design challenge in any NoC-based system and is not unique to the many-core design and is to date, not fully explored in the design of NoC-based hardware for genomic data analysis. Therefore, there is a requirement for new routing schemes, which are tailored for the NoC-based SRA hardware implementations, congestion aware and able to adapt to dynamic traffic requirements. This exploration is done in conjunction with topology design, investigating hybrid and hierarchical topologies such as combining ring, mesh and star, to name a few (Carrillo *et al.*, 2013; Subbulakshmi and Balamurugan, 2014). In addition, some general NoC-based system designs have explored the actual compression of data packets (Carrillo *et al.*, 2012; Maruyama *et al.*, 2017) and prediction of data traffic (Das and Ghosal, 2018; Javed *et al.*, 2020; Maruyama *et al.*, 2017) as a means to accelerate the execution of the application. These design decisions establish the challenge, complexity and motivation, to investigate NoC traffic compression and prediction techniques as mechanisms to advance SRA performances further.

## 6 Conclusion

This review offers a critical analysis of some of the key technical challenges and opportunities within genomic data analysis. SRA is a primary bottleneck due to the volume of raw sequence data for alignment. Various solutions explored throughout offer increased data throughput by scaling the system. As one might assume, this does not ensure effective use of resources. Thus, a point exists where further scale produces diminishing returns on data throughput and acceleration of execution speeds. The use of dedicated external memory to support computational blocks is a convenient way to facilitate this scale and overcome the challenge of memory bandwidth. However, it fundamentally limits the scalability of such solutions, increasing power requirements and financial cost of implementation. This leads to a step backwards from one of the core concepts of personalized medicine, that of being routine and readily available. The use of network architectures to increase the global accessibility of RAM could potentially remove the dependency on dedicated RAM modules per computational block, thereby re-introducing the economy of scale. This review article has attempted to establish future research directions in the utilization of NoCs for SRA hardware acceleration with a focus on combined NoC topology and routing algorithm co-design. In addition, the article has identified the requirements for the co-design of the NoC topology and routing algorithm to accelerate SRA in hardware.

## Funding

This work was supported by the Department for the Economy (DfE), Northern Ireland, UK.

## Conflict of Interest

None declared.
